# Prognostic value of Flotillin-1 expression in patients with solid tumors

**DOI:** 10.18632/oncotarget.17075

**Published:** 2017-04-13

**Authors:** Yang-xi Ou, Fang-teng Liu, Fang-ying Chen, Zheng-ming Zhu

**Affiliations:** ^1^ Department of General Surgery, The Second Affiliated Hospital of Nanchang University, Nanchang 330000, Jiangxi Province, P. R. China; ^2^ Jiangxi Medical College, Nanchang University, Nanchang 330006, Jiangxi Province, P. R. China; ^3^ The Health Centers of Fengzhou Town, Quanzhou 36200, Fujian Province, P. R. China

**Keywords:** Flotillin-1, carcinoma, prognosis, meta-analysis

## Abstract

**Background:**

In numerous studies, Flotillin-1 was reported to be involved in tumor progression, indicating prognosis in various types of cancer. However, the results were inconsistent.

**Results:**

A total of 2473 patients from 13 articles were included. The results indicated that: (1) Patients detected with high expression level of Flotillin-1 protein had a significantly shorter OS (HR =1.64; 95%CI: 1.39-1.88), statistical significance was also observed in subgroup meta-analyses stratified by the cancer type, nationality, detecting method, cutoff value, analysis type, sample size and publication date. (2) Patients with high Flotillin-1 protein expression level had a poorer DFS (HR = 2.49; 95%CI: 1.64-3.35), a worse RFS(HR = 3.26; 95%CI: 1.10-5.43) and a potential shorter PFS(HR = 1.84; 95%CI: 0.81-2.87). (3) The pooled odds ratios (ORs) showed that increased Flotillin-1 level was also related to lymph node metastasis (OR =6.30; 95% CI: 3.15-12.59), distant metastasis (OR =6.02; 95% CI: 1.50-24.06) and more advanced TNM stage (OR =4.69; 95% CI: 2.74-8.03).

**Materials and methods:**

A comprehensive retrieval was performed in multiple databases, including PubMed, Embase, Web of Science and CNKI. The relevant articles were screened for investigating the association between increased Flotillin-1 expression level and prognosis. Additionally, clinicopathological features data was also extracted from these studies.

**Conclusions:**

High expression level of Flotillin-1 protein was correlated with poorer clinical outcome. It might serve as a prognostic biomarker and a potential predictive factor of clinicopathology in various tumors. Further well-designed clinical studies should be performed to verify the clinical utility of Flotillin-1 in human solid tumors.

## INTRODUCTION

Cancer is a major cause of death in the world, there were 14.1 million new cancer cases and 8.2 million cancer deaths occurred in 2012 worldwide [1]. It is well known that earlier diagnosis of cancer can effectively improve prognosis. However, the disease is often clinically silent at an early stage and most tumor biomarkers are not sensitive and specific. Therefore, it is crucial to explore new and efficient biomarkers to predict the prognosis of cancers. Recently, Flotillin-1 protein has considered to be a new prognostic marker for cancers.

Lipid rafts have been reported to be concerned with the development of several malignancies [2]. Therefore, lipid rafts may be a potential therapeutic target formalignant cancers [3]. Flotillin-1, also called reggie-2, is one of the structural proteins of lipid rafts. Flotillin-1 is one member in flotillin proteins family, and it is expressed mostly in brain, lung, placenta and hematopoietic cells [4, 5]. The human Flotillin-1 gene was located on chromosome 6p21.3, consisting of 13 exons, and code a protein of 47 kDa [2]. Although Flotillin-1 is universally expressed, its function has not been fully understood. It showed that Flotillin-1 was involved in cellular signal transduction, endocytosis and cell adhesion [6–8]. Recently, several studies found that Flotillin-1 was closely related to the occurrence and development of tumor [9–10]. For example, Lin *et al*. revealed that Flotillin-1 was highly expressed in breast cancer specimens, and the high expression level of Flotillin-1 was significantly correlated with later clinical staging and poorer patient survival. In addition, proliferation and tumorigenicity of breast cancer cells would be inhibited by the silence Flotillin-1both *in vitro* and *in vivo* [9]. Therefore, Flotillin-1 plays an important role in the occurrence and development of tumor and may serve as a new prognostic marker for cancer.

Several studies have found that high Flotillin-1 expression was associated with shorter overall survival (OS) and disease-free survival(DFS) in various types of cancer [9, 11–12]. However, the reported prognostic value of Flotillin-1 differed in individual original publications. Until now, there was no systematic study on the prognostic value of Flotillin-1 protein in human tumors. Therefore, this meta-analysis was conducted to investigate the prediction value of elevated Flotillin-1 expression level in solid tumors.

## RESULTS

### Study characteristics

**Figure 1:**
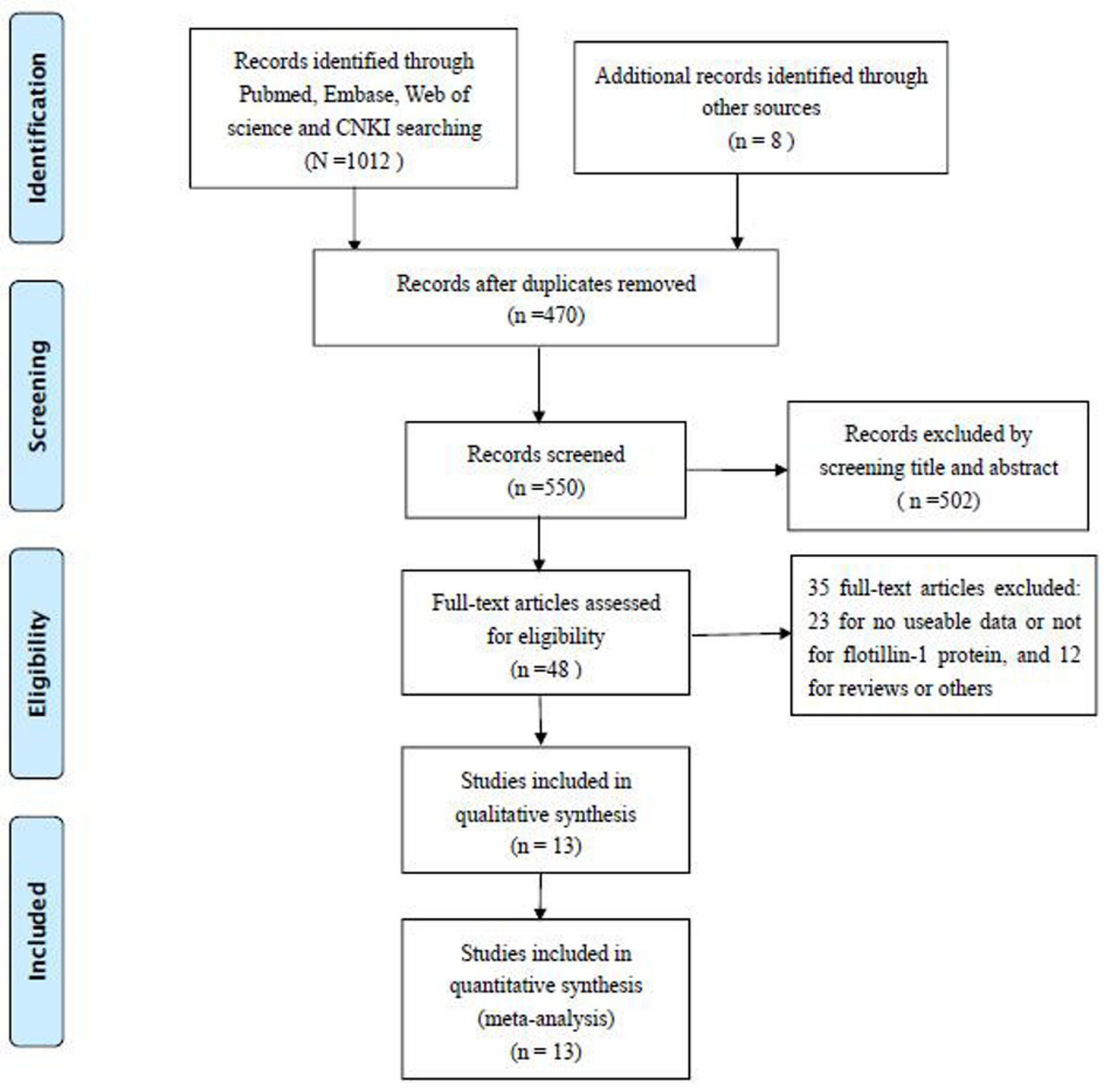
Flowchart presenting the steps of literature retrieval and selection

The detailed process of literature retrieval was shown (Figure 1). According to above mentioned inclusion and exclusion criteria, a total of 13 studies, published from 2011 to 2016, were finally identified as eligible in this meta-analysis [9–21]. The main characteristics of all included studies were summarized (Table 1). There were totally 2473 cancer patients from the People's Republic of China [9–18], Canada [19], Norway [20] and Korea [21]. And the mean patient sample size was 190.2 (ranging from 23 to 432). Nine different kinds of human solid tumors were evaluated in our study, with 3 breast cancer(BC); 1 cervical cancer(CC); 1 esophageal squamous cell carcinoma(ESCC); 1 gastric cancer(GC); 1 hepatocellular carcinoma(HCC); 2 lung carcinoma(LC); 2 renal cell carcinoma(RCC); 1 tongue squamous cell cancer(TSCC) and 1 nasopharyngeal carcinoma(NPC). For the determination of Flotillin-1 expression levels, two assay methods were applied: IHC was applied in 12 studies and WB was applied in 1 study.

**Table 1:** Main characteristics of studies for association between Flotillin-1expression level and human solid cancers

Author, year	Country	Cancer type	Total number, patients (n)	Tumor stage (I/II-III/IV)	Adjuvant therapy beforesurgery	Follow-up (months)	Outcome measures/Analysis type	Detection method	High expression
Lin CY, 2011	China	BC	212	23/97/62/30	*NA*	above 60	OS(M)	IHC	SI≥4
Song LB, 2012	China	ESCC	432	60/194/134/44	*NA*	1-120	OS(M)	IHC	SI≥4
Zhang PF, 2012	China	LC	72	33/6/33 (I/II/III+IV)	NO	1-60	OS(M), RFS(M)	IHC	A combined staining score (extension + intensity) of ≥5
Pust S, 2013	Norway	BC	146	*NA*	*NA*	1-60	DFS(S)	IHC	total score (7-8)
Zhang SH, 2013	China	HCC	196	18/73/102/3	*NA*	median 34.8	OS(M), RFS(S)	IHC	SI≥4
Zhang YY, 2014	China	RCC	182	98/47/23/14	*NA*	above 60	OS(S)	IHC	moderate and strong staining (++ or +++)
Li H, 2014	China	LC	106	66/40 (I + II/III)	NO	1-60	OS(M)	IHC	SI≥4
Li H, 2014	China	TSCC	181	82/67/17/15	NO	above 60	OS(M), PFS(S)	IHC	SI≥6
Cao CL, 2015	China	GC	157	68/89 (I + II/III+IV)	NO	above 60	OS(S), DFS(S)	IHC	SI≥4
Butz H, 2015	Canada	RCC	23	*NA*	*NA*	1-100	OS(S)	Western blot analysis	*NA*
Li Z, 2016	China	CC	308	173/74/28/33 (IB1/IB2/IIA1/IIA2)	NO	above 60	OS(M), PFS(S)	IHC	IRS≥5
Koh M, 2016	Korea	BC	289	92/121/51/0	NO	median 60	DFS(M)	IHC	5% of positive tumor cells with any intensity
Cao SM, 2016	China	NPC	169	13/40/67/49	*NA*	above 60	OS(M), DFS(S)	IHC	SI≥ 8

BC:breast cancer; ESCC:esophageal squamous cell carcinoma; LC:lung carcinoma; HCC:hepatocellular carcinoma;

RCC:renal cell carcinoma; TSCC:tongue squamous cell cancer; NPC:nasopharyngeal carcinoma; CC:cervical cancer;

GC:gastric cancer;

OS:overall survival; RFS:relapse-free survival; DFS:disease-free survival; PFS: progression-free-survival;

IHC:immunohistochemistry; SI:staining index score; IRS: the immunoreactivity score;

(M): Multivariate; (S): Survival curves; *NA*: not available.

### High Flotillin-1 expression and overall survival(OS)

**Figure 2:**
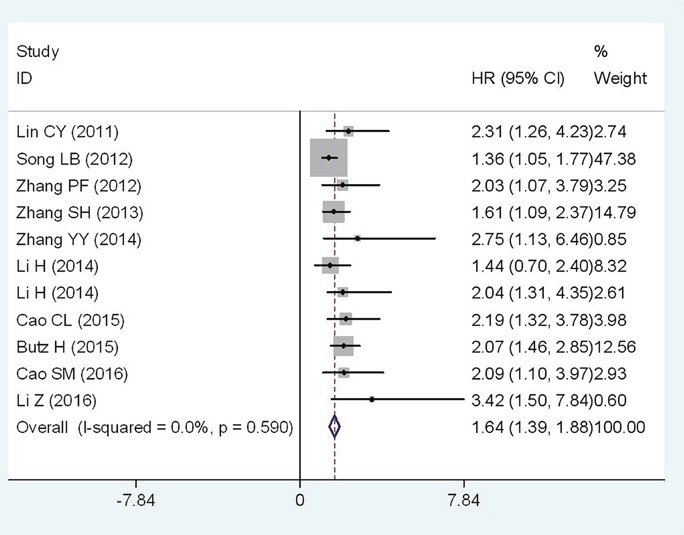
Forest plot of HR for the relationship between high Flotillin-1 expression and OS

A total of 11 studies, including 2038 patients, reported the results of OS towards Flotillin-1 protein expression level in cancerous tissues. Nine kinds of solid tumors were involved for OS, with 1 breast cancer(BC); 1 cervical cancer(CC); 1 esophageal squamous cell carcinoma(ESCC); 1 gastric cancer(GC); 1 hepatocellular carcinoma(HCC); 2 lung carcinoma(LC); 2 renal cell carcinoma(RCC); 1 tongue squamous cell cancer(TSCC) and 1 nasopharyngeal carcinoma(NPC). Because no heterogeneity was observed across-studies (I^2^=0%, P_*h*_=0.590), the fixed-effects model was appliedto pool the HRs. Overall, the meta-analysis showed that high Flotillin-1 expression level was significantly associated with shorter OS (HR=1.64; 95%CI:1.39-1.88, p<0.001) (Figure 2). A worse OS was observed in the patients detected with high Flotillin-1 expressionlevel than those of with low Flotillin-1 expression.

**Figure 3:**
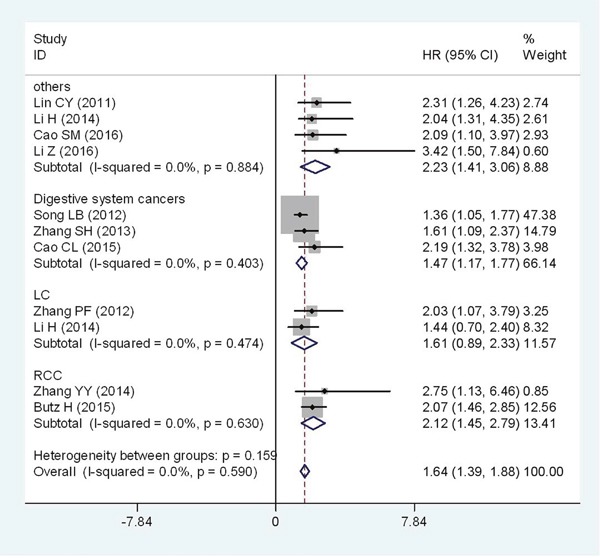
Forest plot of HR for the relationship between high Flotillin-1 expression and OS in various tumors

Further, subgroup analyses were performed according tocancer types. The negative effect of elevated Flotillin-1 on OS was revealed in patients with RCC (HR=2.12; 95%CI: 1.45-2.79, p<0.001) and LC (HR=1.61; 95%CI: 0.89-2.33, p<0.001), and a similar result was also observed in digestive system cancers (HR=1.47; 95%CI: 1.17-1.77, p<0.001) (Figure 3). In addition, for OS, subgroup analyses were performed according to nationality, detecting method, cutoff value, analysis type, sample size, publication date. In those subgroups, meta-analyses, the calculated pooled HR values were significantly larger than 1.0. (Table 2, *those figures were presented in Supplementary Information*).

**Table 2:** Pooled hazard ratios for OS according to subgroup analyses

Categories	Studies (n)	Number of patients	Fixed-effects model		Heterogeneity	
			HR (95% CI) for OS	p-value	I^2^(%)	P_*h*_
[1]OS	11	2038	1.64(1.39-1.88)	<0.001	0.0	0.590
[2]Nationality						
People's Republic of China	10	2015	1.58(1.31-1.84)	<0.001	0.0	0.671
[3]Method						
IHC	10	2015	1.58(1.31-1.84)	<0.001	0.0	0.671
[4]Cutoff value						
SI≥4	5	1103	1.49(1.22-1.77)	<0.001	0.0	0.553
Others	6	935	2.13(1.61-2.64)	<0.001	0.0	0.970
[5]Analysis type						
Multivariate	8	1676	1.53(1.26-1.81)	<0.001	0.0	0.674
Non-multivariate	3	362	2.13(1.54-2.72)	<0.001	0.0	0.886
[6]Sample size						
≥ 100	9	1943	1.56(1.29-1.83)	<0.001	0.0	0.622
<100	2	95	2.06(1.45-2.68)	<0.001	0.0	0.955
[7]Publication year						
2011-2013	4	912	1.49(1.19-1.78)	<0.001	0.0	0.497
2014-2016	7	1126	1.97(1.53-2.40)	<0.001	0.0	0.828
[8]Cancer type						
LC	2	178	1.61(0.89-2.33)	<0.001	0.0	0.474
RCC	2	205	2.12(1.45-2.79)	<0.001	0.0	0.630
Digestive system cancers	3	785	1.47(1.17-1.77)	<0.001	0.0	0.403
Others	4	870	2.23(1.41-3.06)	<0.001	0.0	0.884

### High Flotillin-1 expression and disease-free survival(DFS)

**Figure 4:**
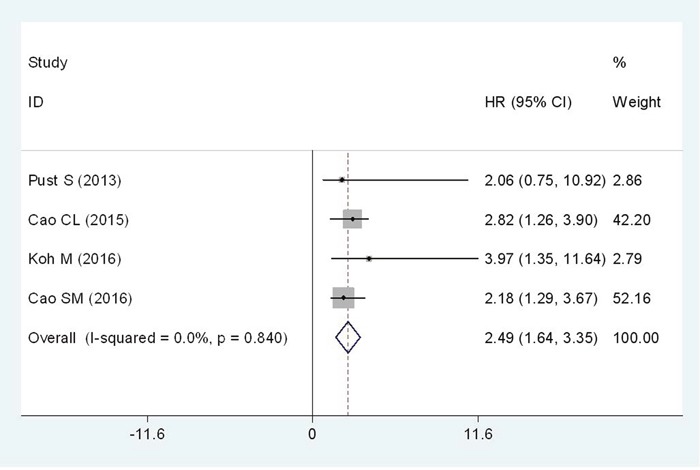
Forest plot of HR for the relationship between high Flotillin-1 expression and DFS

Four studies comprising of 761 patients have investigated the relationship between Flotillin-1 expression level and DFS in cancer patients. Because there was no severe heterogeneity among studies(I^2^= 0%; P_*h*_= 0.840), the fixed-effects model was applied to evaluate the pooled HR with corresponding 95% CI. The pooled result demonstrated a significantly positive association between high Flotillin-1 expression level and poorer DFS (HR = 2.49; 95%CI: 1.64-3.35, p<0.001) (Figure 4).

### High Flotillin-1 expression and relapse-free survival(RFS)

**Figure 5:**
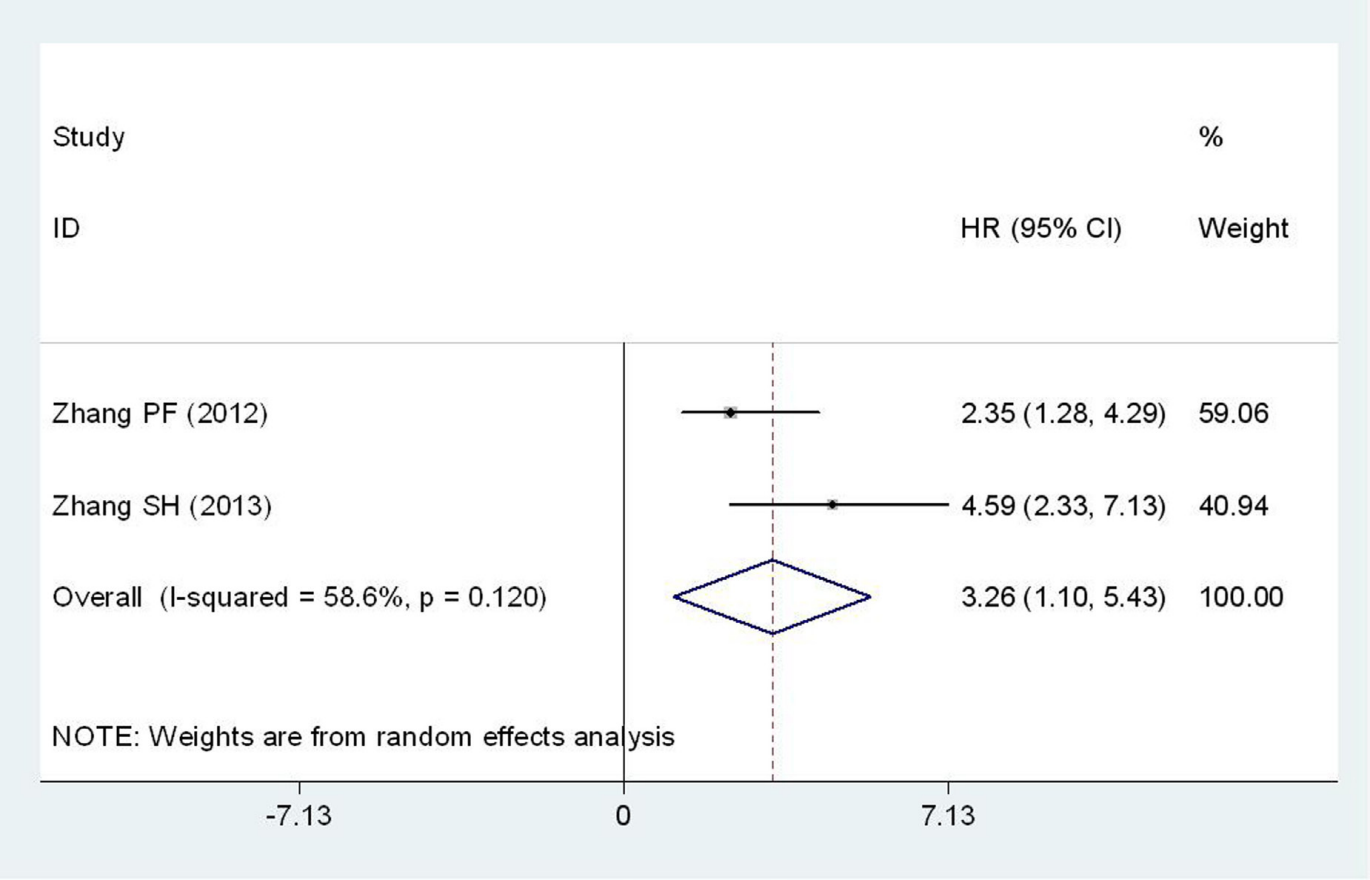
Forest plot of HR for the relationship between high Flotillin-1 expression and RFS

Two studies comprising of 268 patients have provided available information for RFS analysis. For the heterogeneity detected among studies (I^2^=58.6%, P_*h*_=0.120), the random-effects model was applied to analyze the pooled HR with corresponding 95% CI. Patients with low Flotillin-1 expression might had a better relapse-free survival (HR = 3.26; 95%CI: 1.10-5.43, p= 0.003) (Figure 5).

### High Flotillin-1 expression and progression-free-survival(PFS)

**Figure 6:**
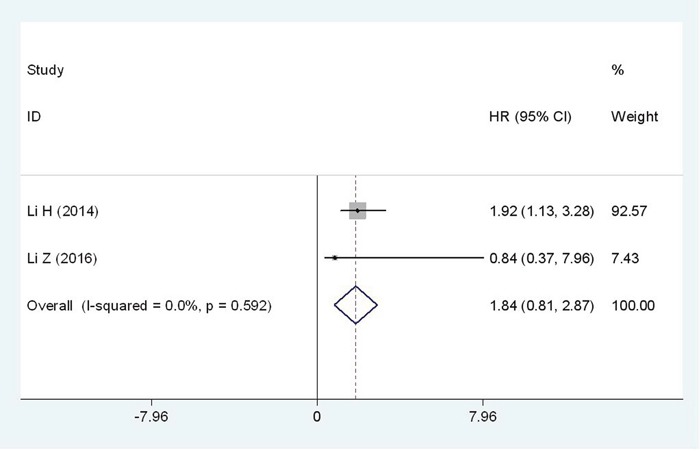
Forest plot of HR for the relationship between high Flotillin-1 expression and PFS

Two studies reported the association between the Flotillin-1 expression level and PFS. A total of 489 patients was included in this meta-analysis. No significant heterogeneity was observed between studies(I^2^=0%, P_*h*_=0.592), therefore the fixed-effects model was employed to estimate the pooled HR with corresponding 95% CI. The pooled results revealed that there may be a negative correlation between the level of Flotillin-1 protein expression and PFS (HR = 1.84; 95%CI: 0.81-2.87, p<0.001) (Figure 6).

### High Flotillin-1 expression and clinicopathological parameters

Meta-analysis indicated that Flotillin-1 expression level was related to histological grade(OR =2.22; 95% CI: 1.04-4.78, p=0.04) and depth of primary tumor invasion(OR =2.73; 95% CI: 1.44-5.17, p=0.002) (Table 3, *All those figures was presented in Supplementary Information*). Additionally, high expressionlevelof Flotillin-1 was also found to be significantly associated with lymph node metastasis (OR =6.30; 95% CI:3.15-12.59, p<0.001), distant metastasis (OR =6.02; 95% CI: 1.50-24.06, p=0.01) and more advanced TNM stage (OR =4.69; 95% CI: 2.74-8.03, p<0.001). However, gender was not significantly related to Flotillin-1 expression. Owing to insufficient data, we failed to detect the relationship between high Flotillin-1 expression and other clinicopathological features.

**Table 3:** Meta-analysis results of the associations of high Flotillin-1 protein expression level with multiple clinicopathological parameters

Clinicopathological parameter	Studies (n)	Number of patients	OR (95% CI)	p-value	Heterogeneity		
					I^2^ (%)	P_*h*_	Model
Sex(male vs. female)	7	1326	0.94(0.73-1.20)	0.61	0	3.45	Fixed effects
Histological grade(poor/moderate vs. well)	7	1542	2.22(1.04-4.78)	0.04	85	39.72	Random effects
T classification (T_3-4_ vs. T_1-2_)	5	1054	2.73(1.44-5.17)	0.002	69	12.98	Random effects
Lymph node metastasis(yes vs. no)	7	1468	6.30(3.15-12.59)	<0.001	84	38.67	Random effects
Distant metastasis (yes vs. no)	3	801	6.02(1.50-24.06)	0.01	68	6.16	Random effects
TNM stage(III-IV vs. I-II)	8	1538	4.69(2.74-8.03)	<0.001	75	27.58	Random effects

### Sensitivity analysis

**Figure 7:**
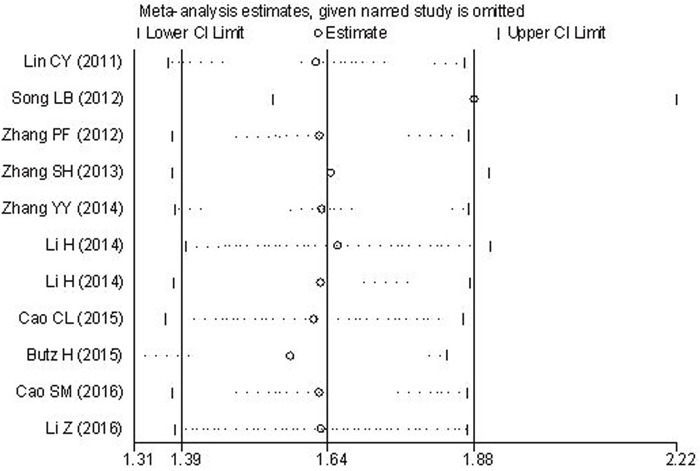
Results of sensitivity analysis for OS

For the association between Flotillin-1 expression level and OS, sensitivity analysis was performed by sequentially omitting each study from the pooled analysis. When any individual study was discarded, the pooled HRs was not significantly changed, suggesting that the results were stable and reliable (Figure 7).

### Publication bias

**Figure 8:**
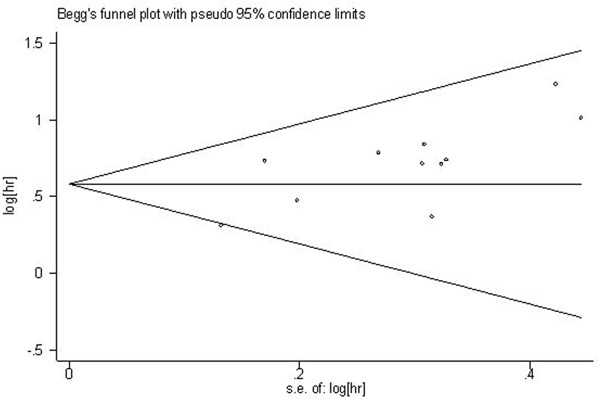
Begg's funnel plot of Flotillin-1 expression and OS

For the meta-analysis of the association between Flotillin-1 expression levels and OS, Begg's funnel plots and Egger's test were applied to assess publication bias. Begg's funnel plot (Figure 8) showed that there was no significant publication bias observed among those studies (Pr > |z| = 0.139, z = 1.48). While the p-value of Egger's test exhibited a slight publication bias among studies (p= 0.011). And then, the trim and fill method was applied to test for publication bias. The results suggested there was no significant publication bias across-studies. Because of the limited number of included studies (n<10), the publication biases for DFS, RFS, PFS and clinicopathological parameters were not assessed.

## DISCUSSION

Flotillin-1 was generally over-expressed in breast cancer, colorectal cancer, liver cancer, esophageal squamous cell carcinoma [22–25] and so on. A new study indicated that Flotillin-1 expression was upregulated significantly in transitional cell carcinomas(TCCs) compared to normal urothelial tissue, moreover, Flotillin-1 expression was significantly associated with tumor size, pathologic grade, clinical stage and recurrence based on t-tests [26]. These findings suggested that Flotillin-1 could be a new target for future cancer immunotherapy. Although Flotillin-1 have been discovered for many years, the function of it in carcinogenesis is not fully clear. According to the review of relevant publications, Flotillin-1 has participated and regulated the tumor development processes with multiple pathways.

Flotillin-1 was critical regulator in receptor tyrosine kinase(RTKs) pathway. Flotillin-1 was involved in signaling processes of RTKs, such as lgE receptor, EGRF and insulin [27–30]. Flotillin-1 could activate EGFR signal pathways by recruiting receptor kinases to lipid rafts [17]. Researchers found that knockdown of Flotillin-1 in MCF7 cells resulted in upregulation of EGFR mRNA and protein expression [31]. Flotillin-1 has made effects with Akt/FOXO3a pathways in breast cancer. The silencing Flotillin-1 would reduce the activity of Akt and then enhance the activity of FOXO3a. Thus, upregulation of cyclin-dependent kinase inhibitor p21(Cip1) and p27(Kip1), and downregulation of the CDK regulator cyclin D1, the proliferation and tumorigenesis of breast cancer cells would be inhibited [32]. Flotillin-1 has participated in the activation of NF-κB pathways. In esophageal squamous cell carcinoma, Flotillin-1 facilitated recruitment of the tumor necrosis factor-α receptor to lipid rafts; promoted K63-linked polyubiquitination of the signaling intermediaries tumor necrosis factor receptor associated factor 2, receptor interacting protein and NEMO; and sustained the activation of NF-κB. Levels of FLOT1 correlated with activation of NF-κB in ESCC samples from patients [33]. These findings suggested that Flotillin-1 would be a key target in integrative tumor development system.

The determination method of Flotillin-1 protein expression would be critical if it would be applied as a prognostic marker. The method for characterizing Flotillin-1 protein expression would be standardized, following factors should be considered. Firstly, the determination method for biomarkers should be unified, no matter IHC, IFA or ELISA. For Flotillin-1 in solid tumor, the IHC was the most common and robust method for its characterization. Secondly, the reagents and controls should be stable and commercially available. The reagents should be provided as kit, including the primary antibody, 2^nd^ antibody and developer. The controls should be also stable for quality control. The cut-off value should be determined per the instruction of the kit. Thirdly, a standard manipulation procedures should be established, such as consistent sample processing, incubation time etc. In addition, automatic device should be involved for more stable results. Specifically, the unified criteria should be verified with a well-designed, large-scale, multi-center case-control study. The result should be further evaluated, including the significance, correlation, corrected cutoff values.

Our meta-analysis provided strong evidence that high Flotillin-1 protein expression was statistically significant associated with shorter OS in patients with solid tumors. The subgroup analysesshowed that the negative effect of elevated Flotillin-1 on OS was revealed in patients with RCC, LC and digestive system cancers. Furthermore, the present meta-analysis suggested that cancer patients with elevated level of Flotillin-1 have a significantly poorer DFS, RFS and potential worse PFS. Our meta-analysis also suggested that flotillin-1 expression level was related to histological grade and depth of primary tumor invasion. Additionally, high expression of Flotillin-1 was also found to be significantly associated with metastasis and more advanced TNM stage. However, gender was not significantly related to Flotillin-1 expression. Our results indicated an important role for Flotillin-1 in the development and progression of tumor, the precise mechanisms of its effects have been demonstrated, however, it should be proved with further well-designed clinical studies.

As we know, this is the first meta-analysis providing precise evidence that elevated expression level of Flotillin-1 protein would be significantly correlated with poor clinical outcomes in patients with solid tumors. However, there are still some limitations in our meta-analysis. First, only 13 studies were included in this study, which resulted in relatively insufficiency data in the subgroup analyses. Especially, only two studies were included in the relationship between Flotillin-1and RFS, PFS. The credibility of results would be reduced. Second, patients included in the meta-analysis were mostly Asians from P. R. China, researches from other countries and races were relatively less. Additionally, publication bias may exist, even though no significant publication bias was observed based on the trim and fill method, as well as stable results were revealed in sensitivity analysis. Finally, different measuring method and cut-off values were applied in those studies, which may affect the availability of Flotillin-1 as a predictive biomarker in cancer prognosis. In view of this situation, further studies with larger sample size are still needed to identify the most appropriate measuring method and cut-off value.

In conclusion, our results clearly suggested that flotillin-1 could be applied for improving prognosis estimation of solid tumors. Considering the limitations of present analysis, this conclusion should be regarded cautiously. Further prospective large-scale, multi-center case-control studies designed adequately with larger sample size are needed to confirm the prognosis value of Flotillin-1 in cancer patients, as well as to explore the function mechanism for guiding effective therapy strategies.

## MATERIALS AND METHODS

### Literature search

For obtaining potentially eligible studies, a comprehensive literature retrieval was conducted in PubMed, Web of Science, Embase, and CNKI, with a cut-off date of Nov. 10, 2016. Only the results from articles in English or Chinese were included. The keywords for the search were as follows: “Flotillin-1”, “reggie-2”, “FLOT-1”, “cancer”, “carcinoma”, “neoplasm” and “tumor”. In addition, the references of other relevant articles were also manually reviewed.

### Inclusion and exclusion criteria

Inclusion criteria for the articles were as follows: (1) the role of Flotillin-1 in the development of human cancer was investigated; (2) associations of Flotillin-1 protein expression with prognosis or clinicopathological features were described; (3) the expression levels of Flotillin-1 protein in primary cancerous tissue were measured; (4) patients were divided into high and low expression groups according to the expression level of Flotillin-1protein; and (5) the articles were written in English or Chinese.

Exclusion criteria for the articles were as follows: (1) duplicate publications; (2) studies without available or usable data; and (3) reviews, letters, case reports and expert opinions.

### Data extraction and quality assessment

Two investigators (Ou YX and Liu FT) independently extracted data and information from the included studies. For any disagreements, the consensus was reached by group discussion. The following data and information were retrieved from each publication: the name of first author, the year of publication, original country, cancer type, number of patients, tumor stage, follow-up period, outcome measures, the criteria for high Flotillin-1 expression, assay methods and hazard ratio (HR) as well as corresponding 95% confidence interval (CI). Besides, the data of clinicopathological parameters were also extracted from the eligible studies. The Newcastle-Ottawa Scale (NOS) was applied to assess the quality of all included studies. The NOS scores ranged from 0 to 9, and the study with an NOS score ≥ 6 was considered to be of high quality. The quality of all studies included in this meta-analysis varied from 6 to 8, with a mean valueof 6.8.

### Flotillin-1 determination

The level of Flotillin-1 protein was determined based on immunoassay. The Flotillin-1 was detected with immunohistochemistry(IHC) in most included studies and it was detected with Western blot(WB) in only one study. For IHC, the staining index score (SI) and immunoreactivity score (IRS) were applied for evaluating the Flotillin-1 level. For WB, the intensity of protein stripe was compared to that of in control protein. For Flotillin-1 assay, the reagents were different with different interpretation criteria. There was also no consensus of cutoff value for determining the high and low expression level of Flotillin-1. In individual study, the cutoff value of Flotillin-1 expression level was determined per their own rules, either based on the instruction of use of reagents manufacturer or clinical experience or existing publications. However, the results in individual study could be comparable for the consistent conditions.

### Statistical methods

Statistical analyses of HRs for prognostic value was calculated by Stata SE12.0 (StataCorp, College Station, TX), and the ORs(odds ratios) for clinical pathological parameters was calculated through RevMan5.3 software. Heterogeneity of pooled results was assessed with Cochrane's Q test and I^2^ measurement. A P value for Q test exceeding 0.05 and/or I^2^ value less than 50 % were considered as no severe heterogeneity, then the fixed effects model was applied; otherwise the random-effects model would be used (P_*h*_≤0.05, I^2^≥50%). Sensitivity analysis was performed to evaluate the validity and reliability of the meta-analysis. Begg's funnel plot and Egger's test were applied to evaluate the publication bias risk. The p-value of less than 0.05 was defined as statistically significant. For studies with both the results of univariate and multivariate analysis, only the latter was selected because of its higher precision as accounting for confounding factors. If a study reported only Kaplan-Meier curves, Engauge Digitizer version 4.1 (http://digitizer.sourceforge.net/, a free down-loaded software) was involved to extract the survival data.

## SUPPLEMENTARY MATERIALS FIGURES


