# Supplementary material for: Prognostic value of Flotillin-1 expression in patients with solid tumors
Source: Oncotarget. 2017 Apr 13;8(32):52665–77. doi: 10.18632/oncotarget.17075 (PMC5581059; doi:10.18632/oncotarget.17075)
Supplement: Supplementary file 1 [file oncotarget-08-52665-s001.pdf]

# Prognostic value of Flotillin-1 expression in patients with solid tumors

## SUPPLEMENTARY MATERIALS

## SUPPLEMENTARY FIGURES

(1) Association between Flotillin-1 protein expression and OS:

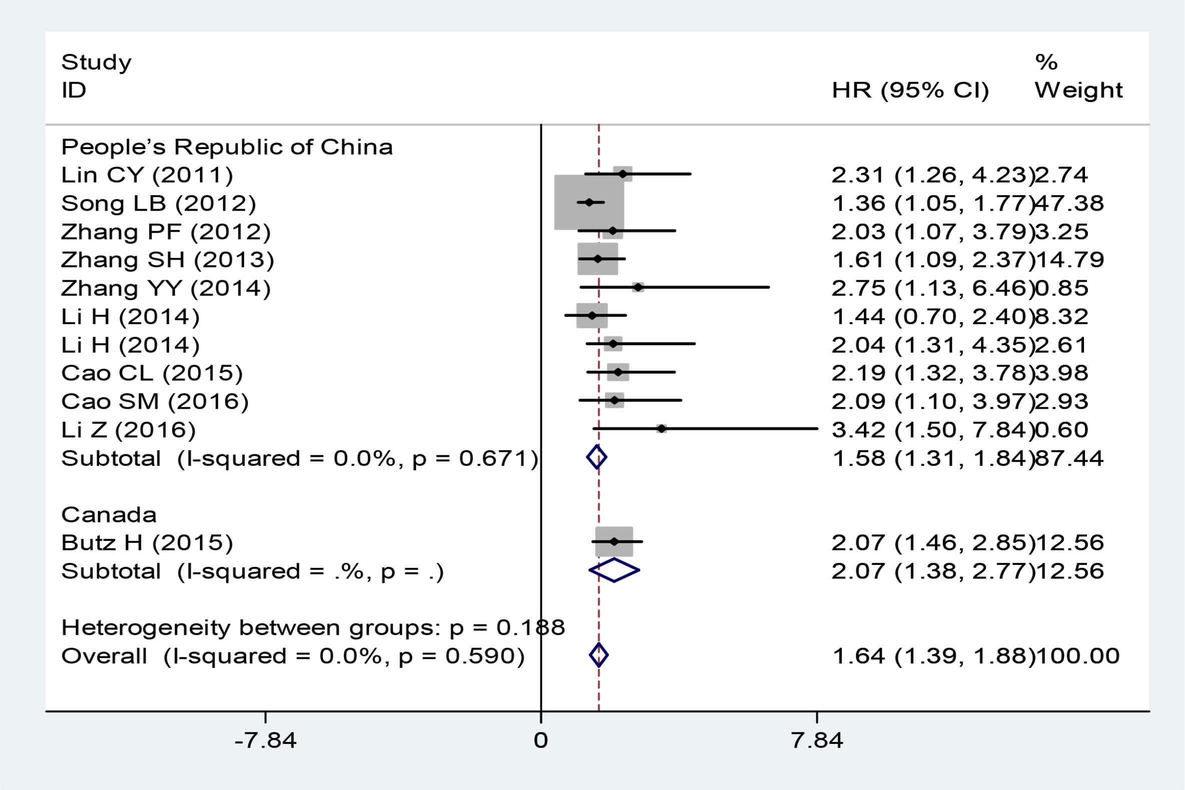


**Supplementary Figure 1: Meta-analysis for the pooled HRs of OS stratified by the nationality.**

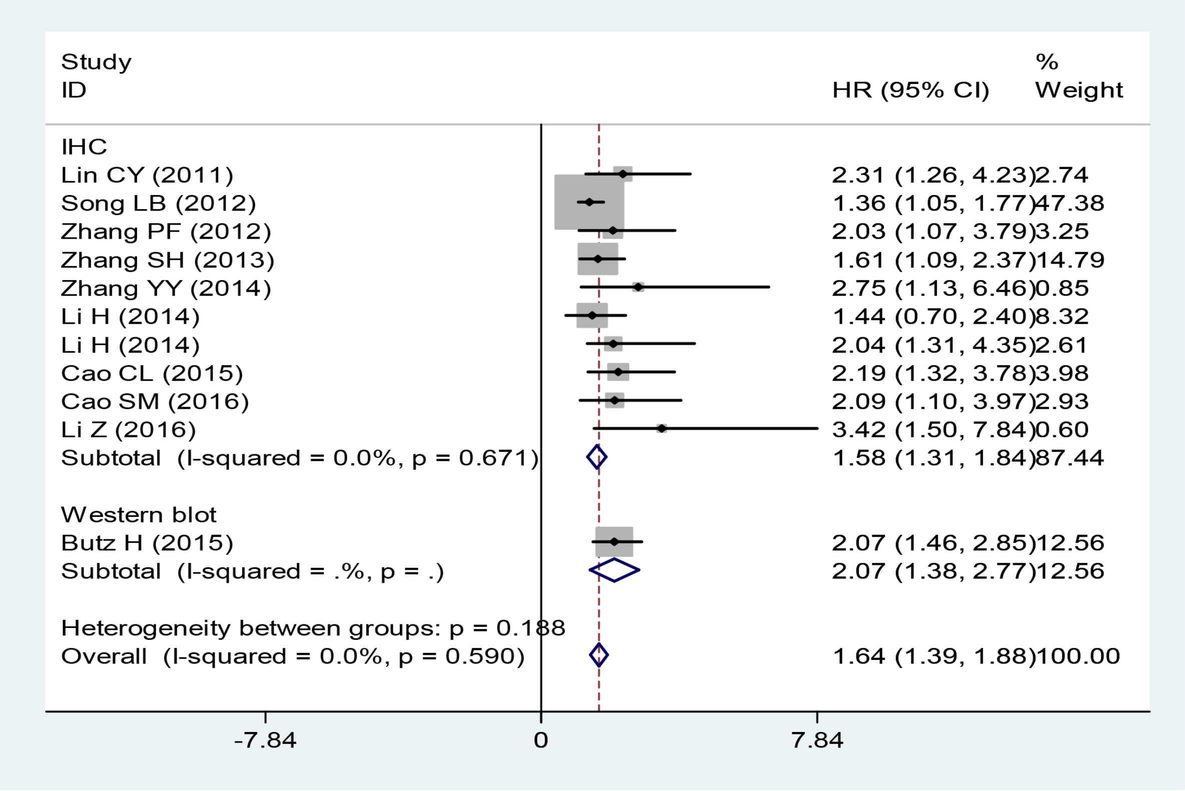


**Supplementary Figure 2: Meta-analysis for the pooled HRs of OS stratified by the detecting method.**

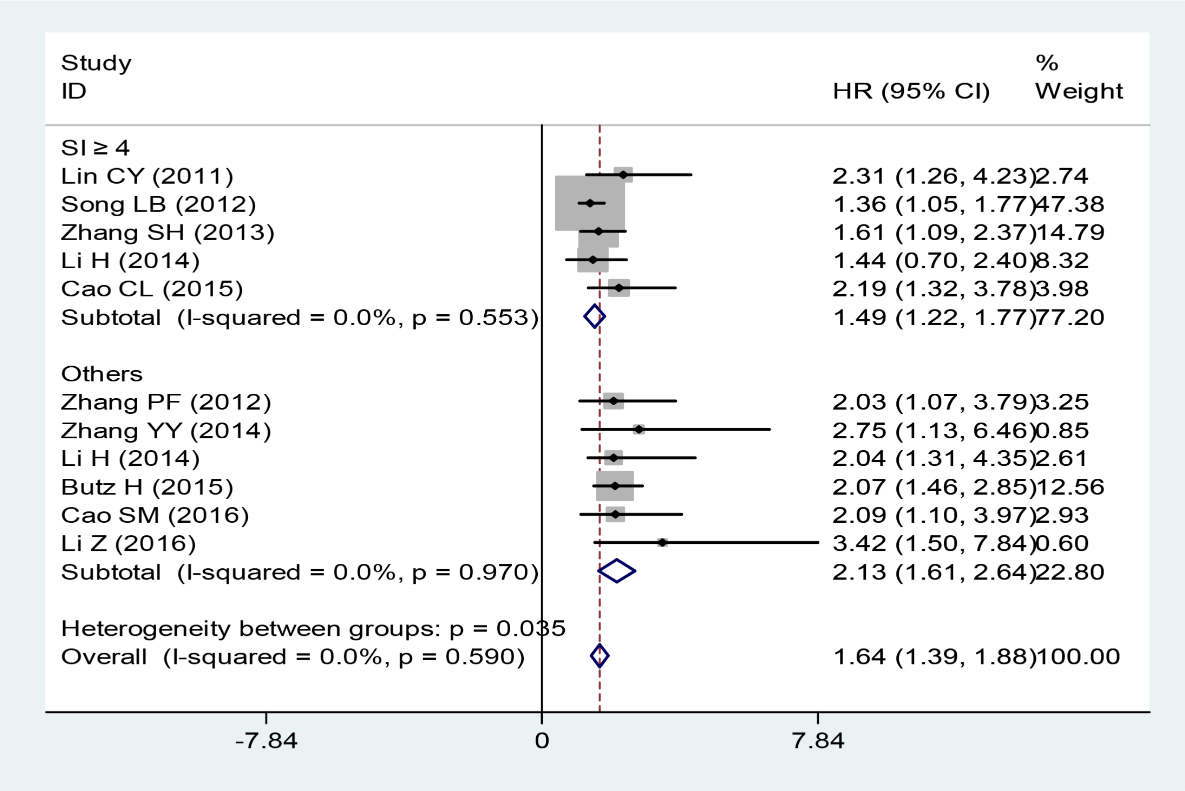


**Supplementary Figure 3: Meta-analysis for the pooled HRs of OS stratified by the cutoff value.**

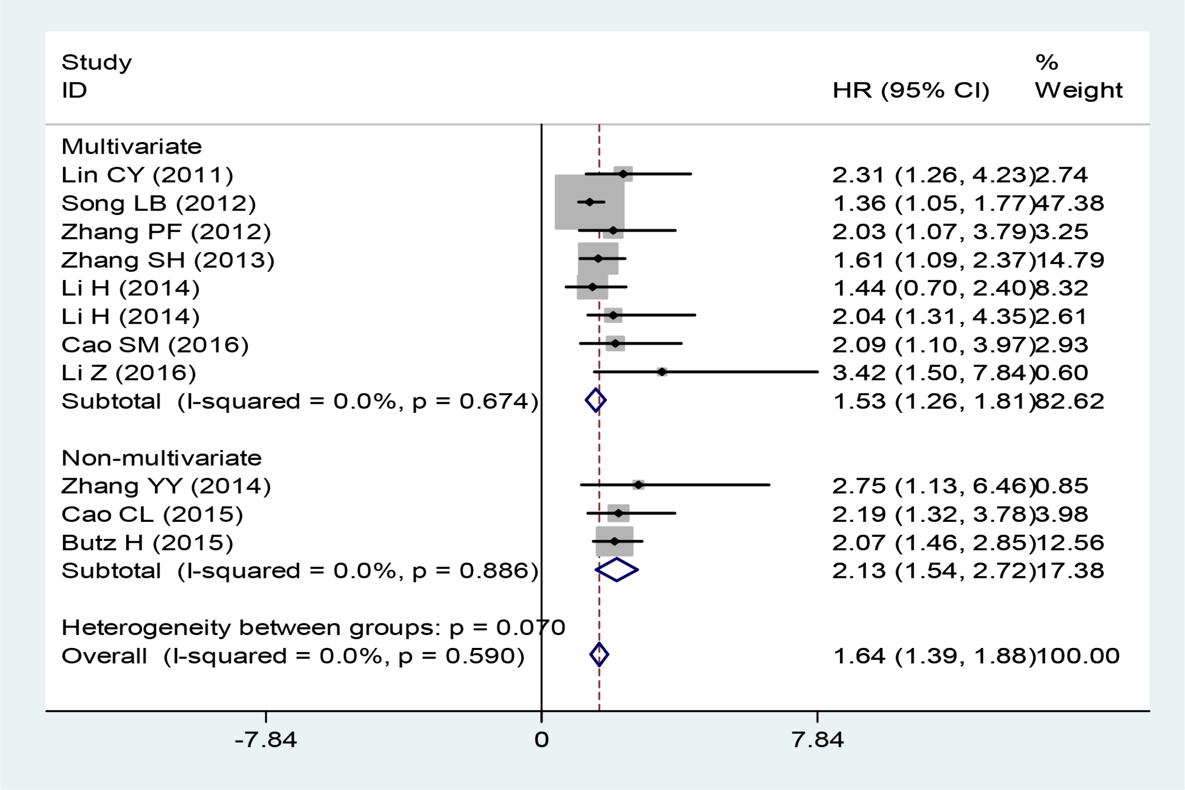


**Supplementary Figure 4: Meta-analysis for the pooled HRs of OS stratified by the analysis type.**

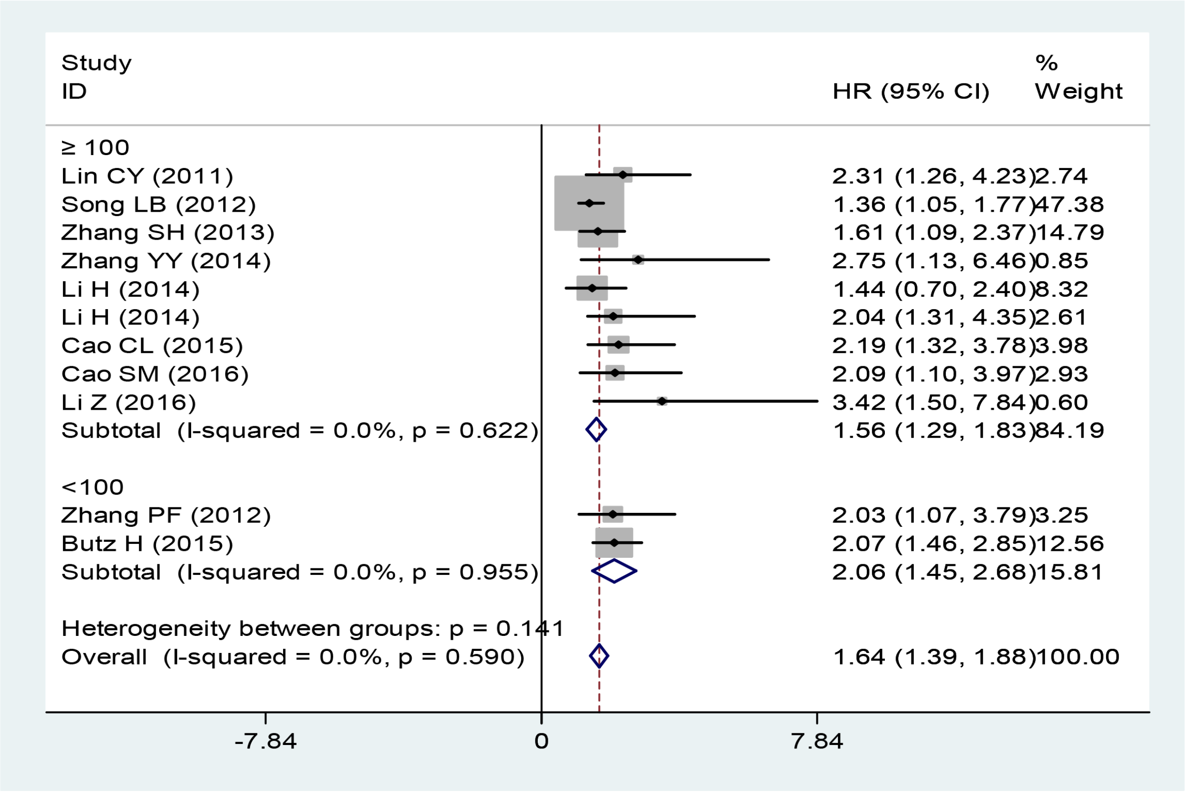


**Supplementary Figure 5: Meta-analysis for the pooled HRs of OS stratified by the sample size.**

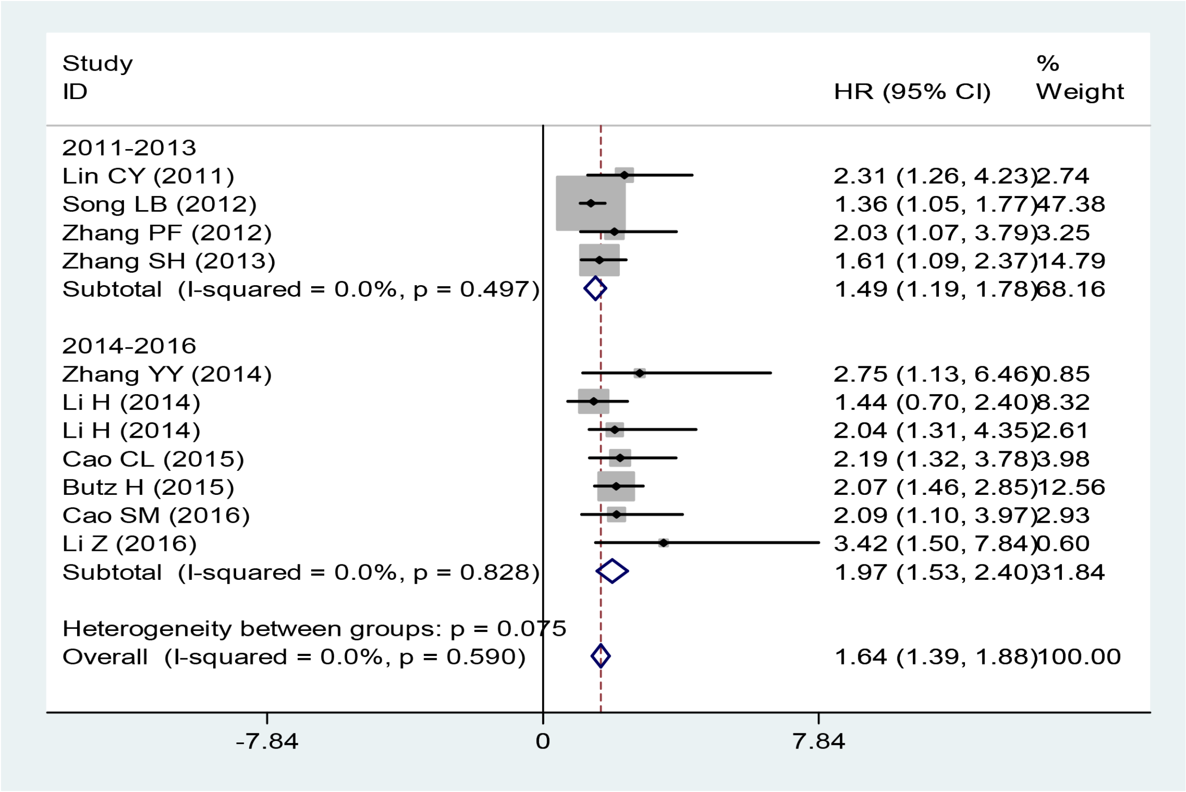


**Supplementary Figure 6: Meta-analysis for the pooled HRs of OS stratified by the publication year.**

(2) Association between Flotillin-1 protein expression and clinicopathological parameters:

| | Male | | Female | | | Odds Ratio | |
|---|---|---|---|---|---|---|---|
| Study or Subgroup | Events | Total | Events | Total | Weight | M-H, Fixed, 95% CI | Year |
| Zhang PF,2012 | 19 | 36 | 17 | 36 | 6.1% | 1.25 [0.50, 3.15] | 2012 |
| Song LB,2012 | 164 | 336 | 50 | 96 | 30.3% | 0.88 [0.56, 1.38] | 2012 |
| Zhang SH,2013 | 88 | 176 | 14 | 20 | 9.6% | 0.43 [0.16, 1.17] | 2013 |
| Li H, -2014 | 53 | 109 | 35 | 72 | 16.5% | 1.00 [0.55, 1.81] | 2014 |
| Li H,2014 | 40 | 74 | 16 | 32 | 7.8% | 1.18 [0.51, 2.70] | 2014 |
| Zhang YY,2014 | 42 | 99 | 37 | 83 | 17.6% | 0.92 [0.51, 1.65] | 2014 |
| Cao CL,2015 | 59 | 85 | 48 | 72 | 12.1% | 1.13 [0.58, 2.22] | 2015 |
| **Total (95% CI)** | | **915** | | **411** | **100.0%** | **0.94 [0.73, 1.20]** | |
| Total events | 465 | | 217 | | | | |

Heterogeneity: Chi² = 3.45, df = 6 (P = 0.75); I² = 0%
Test for overall effect: Z = 0.50 (P = 0.61)

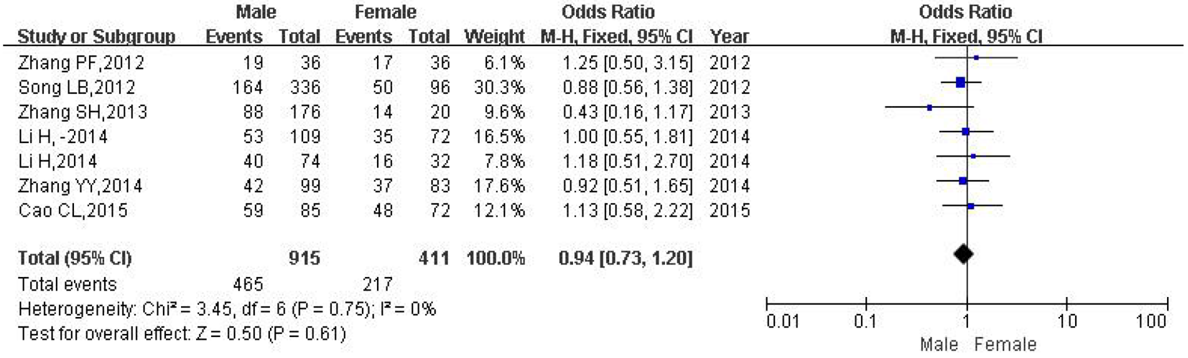


**Supplementary Figure 7: The correlation between Flotillin-1 expression and gender (male vs. female).**

| Study or Subgroup | Poor-moderate Events | Poor-moderate Total | Well Events | Well Total | Weight | Odds Ratio M-H, Random, 95% CI | Year |
|---|---|---|---|---|---|---|---|
| Lin C,2011 | 128 | 200 | 1 | 12 | 7.8% | 19.56 [2.47, 154.57] | 2011 |
| Song LB,2012 | 166 | 298 | 48 | 134 | 17.4% | 2.25 [1.48, 3.43] | 2012 |
| Zhang PF,2012 | 29 | 60 | 7 | 12 | 12.3% | 0.67 [0.19, 2.34] | 2012 |
| Zhang YY,2014 | 62 | 85 | 17 | 97 | 15.9% | 12.69 [6.24, 25.78] | 2014 |
| Li H, -2014 | 24 | 43 | 64 | 138 | 16.0% | 1.46 [0.73, 2.91] | 2014 |
| Cao CL,2015 | 71 | 101 | 36 | 56 | 16.0% | 1.31 [0.66, 2.63] | 2015 |
| Li Z,2016 | 107 | 285 | 9 | 21 | 14.7% | 0.80 [0.33, 1.97] | 2016 |
| **Total (95% CI)** | | **1072** | | **470** | **100.0%** | **2.22 [1.04, 4.78]** | |
| Total events | 587 | | 182 | | | | |

Heterogeneity: Tau² = 0.83; Chi² = 39.72, df = 6 (P < 0.00001); I² = 85%

Test for overall effect: Z = 2.05 (P = 0.04)

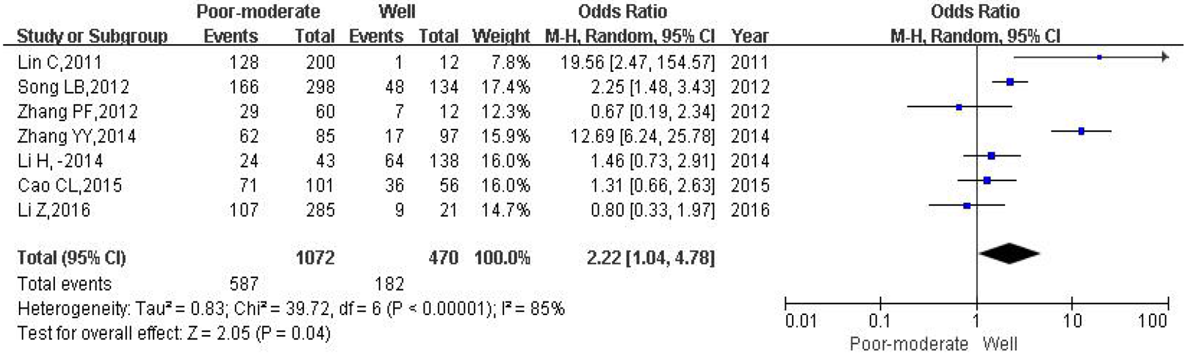


**Supplementary Figure 8: The correlation between Flotillin-1 expression and histological grade(poor/moderate vs. well).**

| Study or Subgroup | T3-4 Events | T3-4 Total | T1-2 Events | T1-2 Total | Weight | Odds Ratio M-H, Random, 95% CI | Year |
|---|---|---|---|---|---|---|---|
| Lin C,2011 | 45 | 61 | 84 | 151 | 24.3% | 2.24 [1.17, 4.32] | 2011 |
| Zhang PF,2012 | 10 | 15 | 26 | 57 | 15.3% | 2.38 [0.72, 7.86] | 2012 |
| Song LB,2012 | 143 | 260 | 71 | 172 | 29.1% | 1.74 [1.18, 2.57] | 2012 |
| Li H, -2014 | 3 | 5 | 85 | 176 | 9.0% | 1.61 [0.26, 9.85] | 2014 |
| Cao CL,2015 | 85 | 101 | 22 | 56 | 22.4% | 8.21 [3.85, 17.50] | 2015 |
| **Total (95% CI)** | | **442** | | **612** | **100.0%** | **2.73 [1.44, 5.17]** | |
| Total events | 286 | | 288 | | | | |

Heterogeneity: Tau² = 0.33; Chi² = 12.98, df = 4 (P = 0.01); I² = 69%

Test for overall effect: Z = 3.08 (P = 0.002)

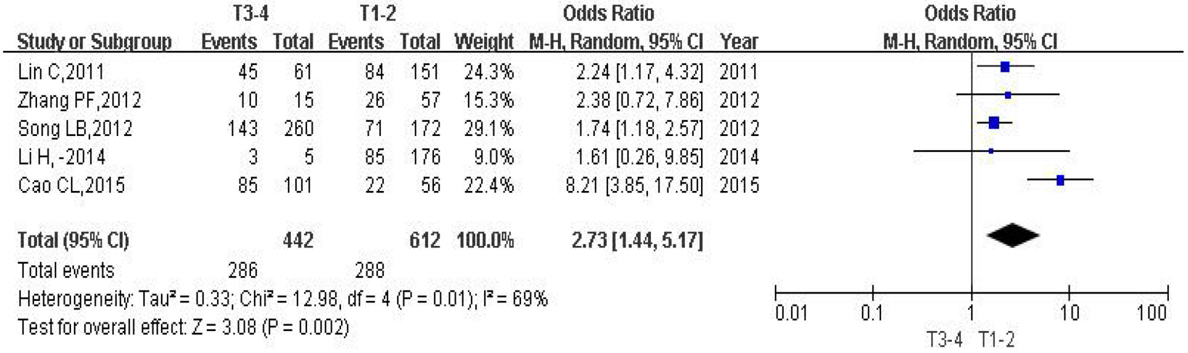


**Supplementary Figure 9: The correlation between Flotillin-1 expression and T classification ($T_{3-4}$ vs. $T_{1-2}$).**

| Study or Subgroup | High expression Events | High expression Total | Low expression Events | Low expression Total | Weight | Odds Ratio M-H, Random, 95% CI | Year |
|---|---|---|---|---|---|---|---|
| Lin C,2011 | 86 | 129 | 39 | 83 | 15.9% | 2.26 [1.28, 3.97] | 2011 |
| Zhang PF,2012 | 32 | 36 | 10 | 36 | 11.2% | 20.80 [5.84, 74.05] | 2012 |
| Song LB,2012 | 137 | 214 | 71 | 218 | 16.9% | 3.68 [2.47, 5.48] | 2012 |
| Li H,2014 | 31 | 56 | 13 | 50 | 14.3% | 3.53 [1.55, 8.04] | 2014 |
| Li H, -2014 | 21 | 88 | 9 | 93 | 14.1% | 2.93 [1.26, 6.81] | 2014 |
| Cao CL,2015 | 101 | 107 | 11 | 50 | 12.6% | 59.68 [20.65, 172.46] | 2015 |
| Li Z,2016 | 39 | 116 | 12 | 192 | 15.1% | 7.60 [3.77, 15.30] | 2016 |
| **Total (95% CI)** | | **746** | | **722** | **100.0%** | **6.30 [3.15, 12.59]** | |
| Total events | 447 | | 165 | | | | |

Heterogeneity: $Tau^2 = 0.70$; $Chi^2 = 38.67$, $df = 6$ ($P < 0.00001$); $I^2 = 84\%$
Test for overall effect: $Z = 5.21$ ($P < 0.00001$)

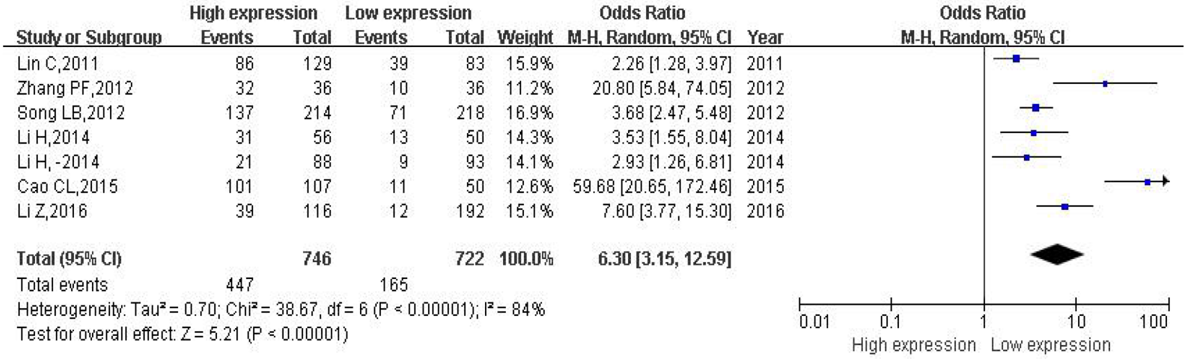


**Supplementary Figure 10: The correlation between Flotillin-1 expression and lymph node metastasis (yes vs. no).**

| | High expression | | Low expression | | | Odds Ratio | |
|---|---|---|---|---|---|---|---|
| Study or Subgroup | Events | Total | Events | Total | Weight | M-H, Random, 95% CI | Year |
| Lin C,2011 | 9 | 129 | 0 | 83 | 16.4% | 13.17 [0.76, 229.32] | 2011 |
| Song LB,2012 | 40 | 214 | 4 | 218 | 41.1% | 12.30 [4.32, 35.04] | 2012 |
| Cao CL,2015 | 25 | 107 | 6 | 50 | 42.6% | 2.24 [0.85, 5.86] | 2015 |
| **Total (95% CI)** | | **450** | | **351** | **100.0%** | **6.02 [1.50, 24.06]** | |
| Total events | 74 | | 10 | | | | |

Heterogeneity: Tau² = 0.93; Chi² = 6.16, df = 2 (P = 0.05); I² = 68%

Test for overall effect: Z = 2.54 (P = 0.01)

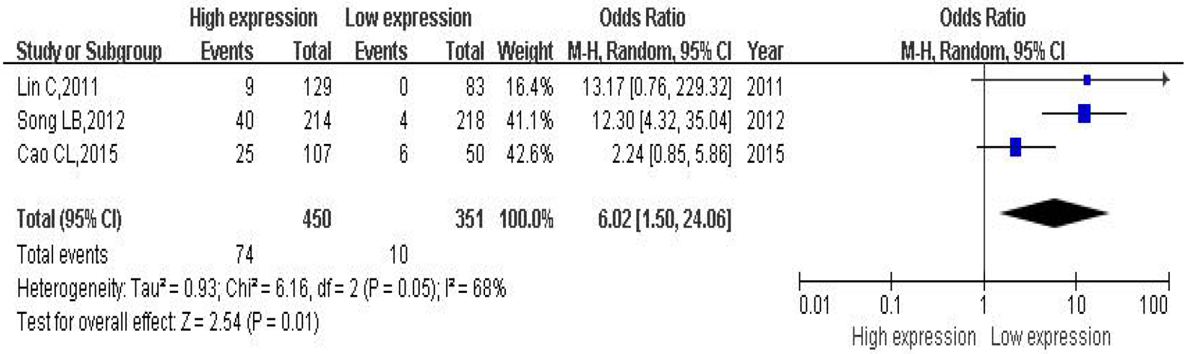


**Supplementary Figure 11: The correlation between Flotillin-1 expression and distant metastasis (yes vs. no).**

| Study or Subgroup | III-IV Events | III-IV Total | I-II Events | I-II Total | Weight | Odds Ratio M-H, Random, 95% CI | Year |
|---|---|---|---|---|---|---|---|
| Lin C,2011 | 68 | 92 | 61 | 120 | 15.6% | 2.74 [1.52, 4.93] | 2011 |
| Song LB,2012 | 129 | 178 | 85 | 254 | 17.2% | 5.23 [3.44, 7.96] | 2012 |
| Zhang PF,2012 | 30 | 33 | 6 | 39 | 7.9% | 55.00 [12.63, 239.56] | 2012 |
| Zhang SH,2013 | 65 | 105 | 37 | 91 | 15.7% | 2.37 [1.34, 4.21] | 2013 |
| Li H, -2014 | 23 | 32 | 65 | 149 | 13.1% | 3.30 [1.43, 7.62] | 2014 |
| Zhang YY,2014 | 37 | 37 | 42 | 145 | 3.1% | 182.65 [10.96, 3042.81] | 2014 |
| Li H,2014 | 29 | 40 | 27 | 66 | 13.0% | 3.81 [1.63, 8.91] | 2014 |
| Cao CL,2015 | 70 | 89 | 37 | 68 | 14.5% | 3.09 [1.54, 6.19] | 2015 |
| **Total (95% CI)** | | **606** | | **932** | **100.0%** | **4.69 [2.74, 8.03]** | |
| Total events | 451 | | 360 | | | | |

Heterogeneity: Tau² = 0.39; Chi² = 27.58, df = 7 (P = 0.0003); I² = 75%
Test for overall effect: Z = 5.64 (P < 0.00001)

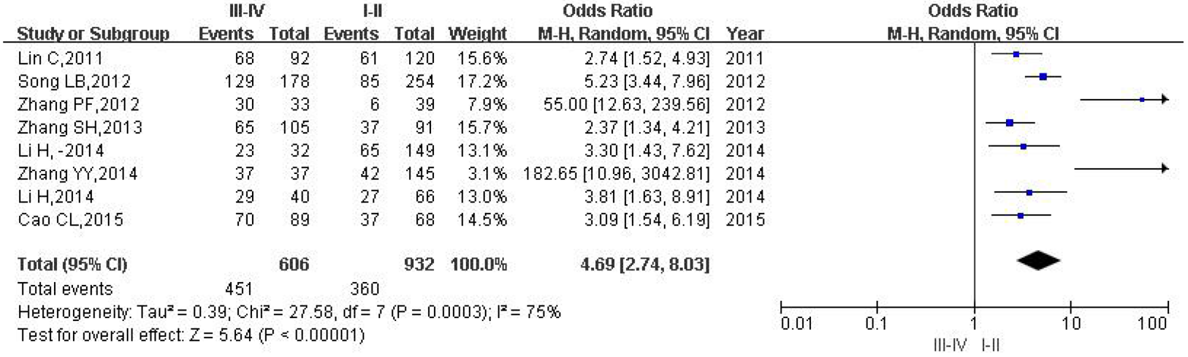


**Supplementary Figure 12: The correlation between Flotillin-1 expression and TNM stage (III-IV vs. I-II).**
